# The Effect of Hormonal Contraceptive Use on Skeletal Muscle Hypertrophy, Power and Strength Adaptations to Resistance Exercise Training: A Systematic Review and Multilevel Meta-analysis

**DOI:** 10.1007/s40279-023-01911-3

**Published:** 2023-09-27

**Authors:** David Nolan, Kelly L. McNulty, Mika Manninen, Brendan Egan

**Affiliations:** 1 School of Health and Human Performance, Dublin City University, Glasnevin, Dublin 9, Ireland; 2 Sport, Health & Exercise (SHE) Research Group, Department of Sport and Health Sciences, Technological University of the Shannon, Athlone, Co. Westmeath Ireland; 3 UPMC Sports Medicine and UPMC Institute for Health, UPMC, Pittsburgh, Ireland; 4 Florida Institute of Human and Machine Cognition, Pensacola, FL USA

## Abstract

**Background:**

Resistance exercise training is widely used by general and athletic populations to increase skeletal muscle hypertrophy, power and strength. Endogenous sex hormones influence various bodily functions, including possibly exercise performance, and may influence adaptive changes in response to exercise training. Hormonal contraceptive (HC) use modulates the profile of endogenous sex hormones, and therefore, there is increasing interest in the impact, if any, of HC use on adaptive responses to resistance exercise training.

**Objective:**

Our aim is to provide a quantitative synthesis of the effect of HC use on skeletal muscle hypertrophy, power and strength adaptations in response to resistance exercise training.

**Methods:**

A systematic review with meta-analysis was conducted on experimental studies which directly compared skeletal muscle hypertrophy, power and strength adaptations following resistance exercise training in hormonal contraceptive users and non-users conducted before July 2023. The search using the online databases PUBMED, SPORTDiscus, Web of Science, Embase and other supplementary search strategies yielded 4669 articles, with 8 articles (54 effects and 325 participants) meeting the inclusion criteria. The methodological quality of the included studies was assessed using the “Tool for the assessment of study quality and reporting in exercise”.

**Results:**

All included studies investigated the influence of oral contraceptive pills (OCP), with no study including participants using other forms of HC. The articles were analysed using a meta-analytic multilevel maximum likelihood estimator model. The results indicate that OCP use does not have a significant effect on hypertrophy [0.01, 95% confidence interval (CI) [− 0.11, 0.13], *t* = 0.14, *p* = 0.90), power (− 0.04, 95% CI [− 0.93, 0.84], *t* =  − 0.29, *p* = 0.80) or strength (0.10, 95% CI [− 0.08, 0.28], *t* = 1.48,* p* = 0.20).

**Discussion:**

Based on the present analysis, there is no evidence-based rationale to advocate for or against the use of OCPs in females partaking in resistance exercise training to increase hypertrophy, power and/or strength. Rather, an individualised approach considering an individual’s response to OCPs, their reasons for use and menstrual cycle history may be more appropriate.

**Registration:**

The review protocol was registered on PROSPERO (ID number and hyperlink: CRD42022365677).

**Supplementary Information:**

The online version contains supplementary material available at 10.1007/s40279-023-01911-3.

## Key Points

**Table:** 

When comparing OCP users to non-users, OCP use has no significant effect on skeletal muscle hypertrophy, power or strength adaptations in response to resistance exercise training.
Based on the present analysis, there is no evidence-based rationale to advocate for or against the use of OCPs in females partaking in resistance exercise training to increase hypertrophy, power and/or strength.
To date, studies investigating the influence of HCs on adaptations to resistance exercise training have exclusively investigated OCPs, and future research should also examine the potential influence of different HC types.

## Introduction

Resistance exercise training (RET) is strongly encouraged for the general population because of its myriad of associated health benefits [1] and is widely used by athletic populations as part of a comprehensive athletic development training program [2]. Resistance exercise training elicits morphological (i.e. increased muscle fibre/whole muscle cross-sectional area, change in muscle fibre pennation angle and increases in the proportion of non-contractile tissues) and neurological (i.e. increased motor unit activation, firing frequency and synchrony of high threshold unit) adaptations which contribute to changes in skeletal muscle hypertrophy, power and strength [3]. Higher levels of muscle strength are associated with superior force–time characteristics (e.g., rate of force development and increased external mechanical power), general sport-related skill performance (e.g., jumping, sprinting and change of direction) and a decreased risk of injury [4].

Hormonal contraceptives (HCs), which involve the administration of exogenous sex hormones that affect endocrine regulation of the female reproductive system [5, 6], are used by a sizeable proportion of individuals in both general (~ 28–43%) [7, 8] and athletic (~ 40–51%) [9–11] populations. HCs are classified according to the hormones employed; combined HCs have both oestrogenic and progestin components, whereas other HCs have a progestin-only component. HCs are also administered using various delivery methods, with the oral contraceptive pill (OCP) being the most commonly used form among young females [8, 12]. Combined OCPs reduce endogenous concentrations of 17-beta oestradiol and progesterone (compared with the mid-luteal phase of the menstrual cycle), acting via negative feedback on the gonadotrophic hormones, chronically downregulating the hypothalamic–pituitary–ovarian axis [12]. Dependent on if, and how, the dosages of exogenous hormones vary across the OCP cycle, the combined OCPs can be monophasic (i.e. consistent dosage), biphasic (i.e. two levels of dosage) or triphasic (i.e. three levels of dosage), and are also classified by “generation”, categorised by the form of progestin used [13].

Endogenous sex hormones influence various bodily functions and may also influence exercise performance [14]. HC use has equivocal effects on acute measures of athletic performance [12], yet the majority of literature to date is of low quality, with small sample sizes, lack of standardisation and inadequate familiarisation, among the important issues that limit interpretation. Relatedly, the impact of HC use on adaptive responses to resistance exercise training has been the subject of increasing interest, with positive (molecular markers) [15], negative (hypertrophy, strength, inflammation) [16–18] and neutral (hypertrophy, strength, power) [19–25] outcomes being observed in HC users compared with non-users. Lack of consistent findings on the influence of exogenous hormones on resistance exercise training adaptations contributes to cause confusion in females and those that work with them, when trying to make an informed decision on whether or not HC is likely to impact athletic performance and/or training adaptations. Given the mixed findings to date, and absence of evidence-based recommendations exist for sportswomen and practitioners who work with them, this review aimed to investigate the influence of HCs on skeletal muscle hypertrophy, power and strength adaptations in response to resistance exercise training.

## Methods

### Literature Search and Management

All items in this protocol correspond with the Preferred Reporting Items for Systematic Review and Meta-Analysis Protocols Statement (PRISMA-P; [26]; see Electronic Supplementary Material Table S1) The review protocol was registered on PROSPERO (ID number and hyperlink: CRD42022365677) on 3 December 2022. The literature used in this meta-analysis was obtained before 6 July 2023 from the following databases: PUBMED, SPORTDiscus, Web of Science and Embase. The first author (DN) gathered the literature from the databases using the following search string for all databases: *(“contraceptive” OR “contraceptives” OR “hormonal” OR “birth control”)AND (“exercise” OR “resistance training” OR “resistance exercise training” OR “hypertrophy training” OR “weightlifting” OR “bodybuilding” OR “athletic training” OR “strength training” OR “power training” OR “plyometric training” OR “jump training” OR “physical training”) AND (“strength” OR “hypertrophy” OR “mobility” OR “power” OR “sprint” OR “rate of force development” OR “RFD” OR “speed” OR “jump” OR “stiffness” OR “reactive strength index” OR “dynamic strength index” OR “flexibility” OR “RSI” OR “DSI” OR “EUR” OR “eccentric utilisation ratio” OR “eccentric utilization ratio” OR “tendon” OR “ligament”).*

**Fig. 1:**
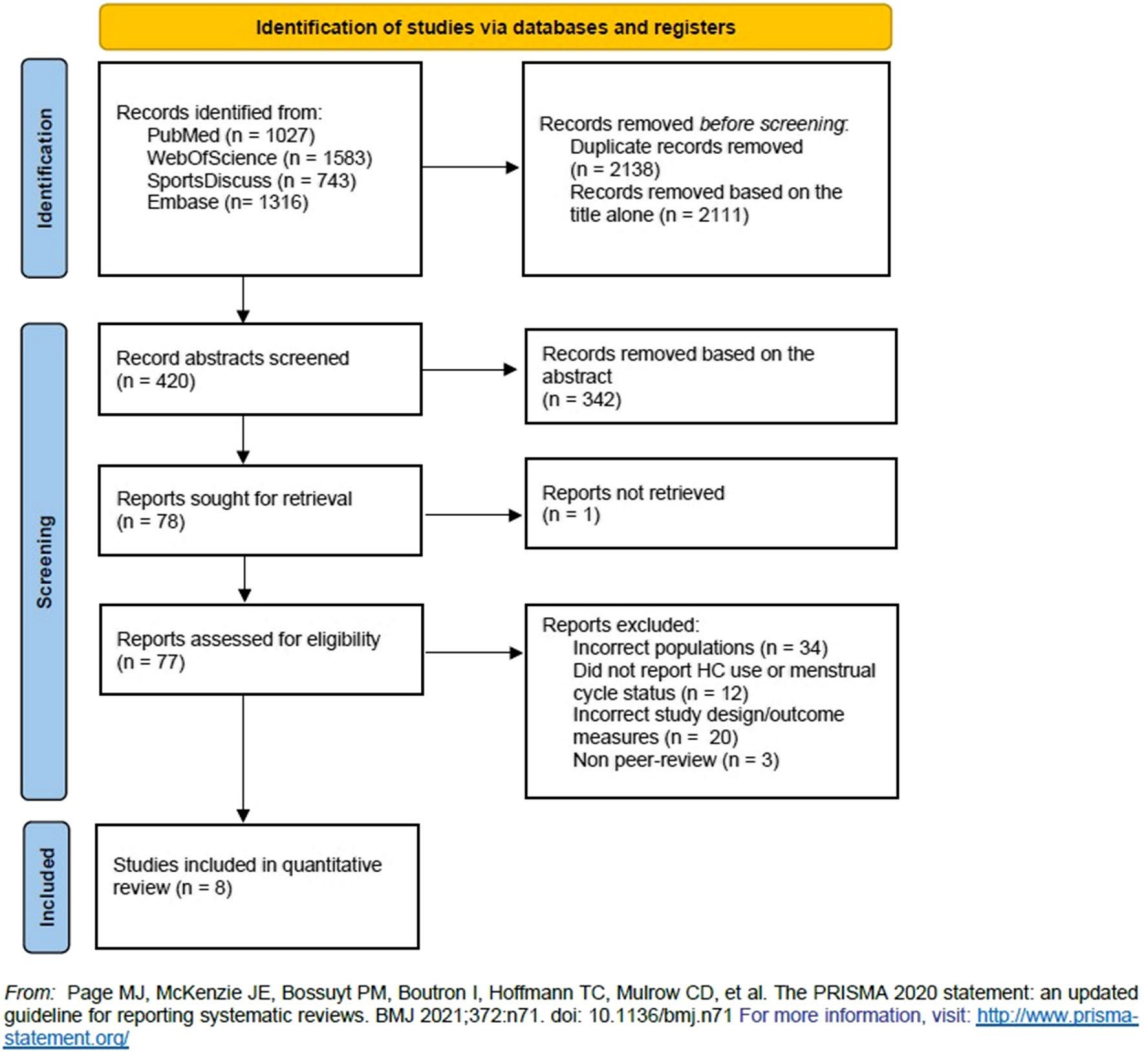
PRISMA flow diagram. Detailed flow of studies examined from the initial search to the final inclusion

In addition to the database search, the reference lists of all the included studies and relevant review studies found in the search were assessed. Moreover, a backward search using Google Scholar was conducted for all included studies. All duplicate articles were removed. The first two authors (DN and KLM) independently assessed each article identified from the searches by applying the exclusion and inclusion criteria to the titles and abstracts. Each study carried forward from this stage was fully read and reviewed independently by these same authors, aiming to determine the studies to be included in the meta-analysis. Conflicting opinions were resolved via discourse between the first and second authors (DN and KLM), with the last authors (BE and MM) acting as mediators, if necessary. Reasons for exclusion of studies were recorded and are displayed in Fig. 1.

### Study Selection

Research publications were considered eligible if the following inclusion criteria were met: (1) all research made available prior to 6 July 2023; (2) were in English and peer-reviewed; (3) were experimental in design; (4) used a resistance exercise training intervention (resistance exercise training was defined as interventions in which the muscles contract against an external resistance with the intent of inducing adaptations resulting in increases in hypertrophy, power or strength); (5) measured muscular hypertrophy, power or strength outcomes; (6) used at least two data points (pre- and post-measures); (7) included healthy biological female participants with a mean age of ≥ 18 and ≤ 40 years; (8) used training interventions ≥ 4 weeks in duration; and (8) had direct comparison of HC users and non-users. The exclusion criteria were as follows: (1) age < 18 or > 40 years; (2) individuals with menstrual dysfunction or other comorbidities; (3) concurrent exercise training interventions; and (4) used training interventions ≤ 4 weeks in duration.

### Data Extraction, Moderators and Study Quality

The first two authors independently extracted sample sizes, means and standard deviations (SD) or standard errors (SE) of the outcome measures from each study. Where data were not reported in sufficient detail, or did not allow for appropriate extraction, requests for data were made by contacting the corresponding author. The authors were contacted a maximum of three times with a 1-week time interval between contact efforts. If the email address of the author was not working or was not publicly available, the private message function of the Research Gate website was used as the method of contact. In one study [22], statistical information was extracted from study figures using WebPlotDigitizer (Version 4.6, Pacifica, CA, USA). Two studies [15, 19] provided data from the same participants and intervention, as confirmed via direct communication with the corresponding author. Thus, the extracted data were considered to be from one study for the purpose of analysis. All described techniques were applied when we did not receive missing information from the study authors, as suggested by the Cochrane Handbook [27]. Inter-rater agreement for all extracted data used in the effect size calculation was assessed using an intraclass correlation coefficient (ICC) for continuous data. Any dissimilarities were located and resolved before the final calculations were completed.

In addition to quantitative information, a priori moderators were extracted, including characteristics of the experimental interventions (duration of intervention, supervision status, mode of resistance training, number of exercises, training frequency, number of sets, intensity and rep ranges used), participant characteristics (age, height, body mass and training status) and features of the paper (country, publication year and research group). Inter-rater agreement for all coded moderators was assessed as an unweighted Cohen’s kappa. Dissimilarities, if any, were located and resolved before the final calculations were completed.

The methodological quality of the included studies was assessed using the “Tool for the assessment of study quality and reporting in exercise” (TESTEX) [28]. TESTEX is a 12-item scale divided into two sections: study quality (Items 1–5) and study reporting (Items 6–12), and represents a modified version of the Physiotherapy Evidence Database (PEDro) scale [29]. The scale was modified for use in this review (Electronic Supplementary Material Table S2). Items 2, 3 and 10, referring to randomisation of intervention groups, allocation concealment and activity monitoring in control groups, respectively, were deemed irrelevant to the design of the studies in this present review and were removed. Two additional items (Items 2 and 10 in the modified version) were included: Q2. Were the participants confirmed to be habitual HC users or habitual non-users for at least 3 months prior to the study? Q10. Was the type of HC described to the level of detail required for categorisation or replication? Each question was awarded one point if the criteria were satisfied, with Items 5 and 7 containing three and two questions, respectively. The maximum number of points that could be scored on this modified 11-item checklist was 14.

### Calculation of Effect Sizes

All the outcomes were analysed as differences between mean change difference (Hedge’s *g*) in response to the training intervention between the HC and non-HC conditions using the escalc function in the metafor package (Viechtbaur, 2010) in R (version 4.0.5; R Core Team, 2022). Standardised mean change for the HC and non-HC conditions were computed using the pre-test standard deviations and a bias correction factor [30]. As the pre–post-test correlations were not available in the studies, an estimate correlation of 0.7 was used to compute the standardised mean changes, while also testing alternative correlations of 0.5 and 0.9. The difference in the standardised mean changes were then computed by subtracting the standardised mean change of the HC condition from the non-HC condition [31]. The corresponding sampling variances were computed by summing the sampling variances of the two conditions.

### Statistical Analysis

A multilevel maximum likelihood random effects model [30] was fitted to the data using R (version 4.0.5; R Core Team, 2022) and the Metafor package (Viechtbauer, 2010). The adopted meta-analytic approach utilised multilevel modelling to account for the non-independence of effect sizes. Specifically, the authors implemented a meta-analytic multilevel model that incorporated a variance–covariance matrix in the model [30]. This approach allowed the authors to account for the fact that the effects sizes were nested within studies, which in turn improved the ability to estimate the true effect size. The models used an estimate of 0.9 for dependence of effects, informed by expert opinion of the authors. As the exact magnitude of dependence of the effects was unknown, robust variance estimator from the clubSandwich package was used to improve the accuracy of the estimates [32].

In the multivariate model, random effects were added for each effect size within each study, allowing the effect sizes to correlate and have different variances. Parameters of *tau2* and *I2* were used to examine the between-study heterogeneity of the effects [33]. Furthermore, as the *Q* statistic for heterogeneity cannot be applied to multilevel models, a likelihood ratio test examining the effect of *tau2* on all outcomes was used as an indicator of significant between-study heterogeneity. The between-study heterogeneity of the effect sizes was indicated if the likelihood ratio test (*χ*^2^) reached a significance level of *p* < 0.05, and the sampling error contributed to the observed variance of less than 75% [34].

The moderators were used in a linear regression analysis as univariate independent variables to explain the possible heterogeneous effects of the outcomes. Interactions of the moderators were not tested because of the lack of statistical significance of the models, low between-study heterogeneity, and inadequate number of effects for certain outcomes (i.e. power) [35]. A modified version of Egger’s test [36] using the standard error of the observed outcomes as a predictor in a multivariate model and a visual examination of the contour-enhanced funnel plots were used to detect publication bias (Electronic Supplementary Material Fig. S1). The presence of outliers and influential studies/effects were analysed using Cook’s distance and the distribution of studentized residuals [37].

The aggregated dataset and R-code used for the analysis can be found on the OSF website (https://osf.io/wumav/?view_only=8ed6db48dcad465ba36fcc95fb6d3ee7). Additional information can be shared on request.

## Results

In total, 54 effects from eight studies were derived for hypertrophy (*k* = 20), power (*k* = 8) and strength (*k* = 26) outcomes. The study selection process from the initial search to final inclusion is shown in Fig. 1. The complete descriptive information of the included studies is presented in Table 1 and Electronic Supplementary Material Table S3. The OCP type and menstrual cycle status of the participants of the included studies are presented in Table 2. The total number of participants was 325 (*n* = 159/166; OCP/naturally-menstruating), with a weighted mean age of 24.0 years. All the included studies investigated the influence of OCPs, with no study including participants using other types of HC. The exercise interventions lasted between 8 and 16 weeks, with a weighted mean duration of 11.6 ± 2.2 weeks, and a weighted mean number of 3.3 ± 0.4 sessions per week. The mean TESTEX scale score was 9.4, with individual studies ranging from 5 to 13. Individual scores for quality assessment can be found in the Electronic Supplementary Material Table S3.

**Table 1:** Characteristics of included studies comparing oral contraceptive pill (OCP) users and OCP non-users following matched resistance exercise training interventions

Study	*n*	Training status	Intervention duration (weeks)	Supervised training	Outcomes	Results OCP versus non-users	Study conclusion	Methodological quality score
Dalgaard et al., 2019 [20]	OCP-users: 14 Naturally menstruating: 14	Untrained	10	Yes	**Hypertrophy:**		Use of OCPs was associated with a trend towards a greater increase in muscle mass and a significantly greater increase in type I muscle fibre area compared with controls. Use of OCPs did not influence the overall increase in muscle strength related to training	12
					MRI:			
					Vastus lateralis muscle CSA	↔		
					Biopsy:			
					Vastus lateralis type I fibre CSA	**↑**		
					Vastus lateralis type II fibre CSA	↔		
					**Strength:**			
					1RM:			
					Knee extension	↔		
					Isokinetic dynamometer IMVC:			
					Knee extension	↔		
Dalgaard et al., 2022* [19]	OCP-users: 20 Naturally menstruating: 18	Untrained	10	Yes	**Hypertrophy:**		The use of a second-generation OCP in young untrained women does not promote significantly greater gains in muscle mass or muscle strength compared with non-users	13
					DXA			
					Fat-free mass	↔		
					MRI:			
					Vastus lateralis muscle CSA (10 cm)	↔		
					Vastus lateralis muscle CSA (20 cm)	↔		
					Vastus lateralis muscle CSA (30 cm)	↔		
					Biopsy:			
					Vastus lateralis type I fibre CSA	↔		
					Vastus lateralis type II fibre CSA	↔		
					**Power:**			
					Counter-movement jump	↔		
					**Strength:**			
					5RM:			
					Leg press	↔		
					Isokinetic dynamometer IMVC:			
					Knee extension	↔		
					Knee flexion	↔		
Nichols et al., 2008 [22]	OCP-users: 13 Naturally menstruating: 18	Trained (NCAA Division 1 Collegiate Athletes)	12	NR	**Strength:**		The use of OCPs did not provide sufficient androgenic effect to increase strength gains beyond the stimulus of the training protocol	6
					1RM:			
					Bench press	↔		
					10RM:			
					Knee extension	↔		
					Isokinetic dynamometer peak torque:			
					Bench press	↔		
					Knee extension	↔		
Oxfeldt et al., 2020* [15]	OCP-users: 20 Naturally menstruating: 18	Untrained	10	Yes	**Hypertrophy:**		Use of second-generation OCPs in young untrained women increased skeletal muscle MRF4 expression and satellite cell number compared with non-users	13
					Biopsy:			
					Vastus lateralis type I fibre CSA	↔		
					Vastus lateralis type II fibre CSA	↔		
Reichmann and Lee, 2021 [16]	OCP-users: 34 Naturally menstruating: 38	Untrained	10	Yes	**Hypertrophy:**		OC use impairs muscle gains in young healthy untrained women, but the effect may depend on the type of OCPs	9
					Hydrostatic weighing:			
					Lean mass	**↓**		
					**Strength:**			
					1RM:			
					Arm strength (aggregated score)	↔		
					Leg strength (aggregated score)	↔		
Romance et al., 2019 [23]	OCP-users: 12 Naturally menstruating: 11	Trained (> *2 years’ continuous resistance exercise training*)	8	Yes	**Hypertrophy:**		OC use does not impair strength gains nor body composition in resistance-trained young adult women	9
					DXA:			
					Fat-free mass	↔		
					**Power:**			
					Counter-movement jump	↔		
					**Strength:**			
					1RM:			
					Bench press	↔		
					Squat	↔		
Sung et al., 2022 [24]	OCP-users: 34 Naturally menstruating: 40	Untrained	12	Yes	**Hypertrophy:**		The effects of RET on muscle strength, muscle thickness, muscle fibre size and composition were similar in young women irrespective of their OCP use	8
					Ultrasound:			
					Muscle thickness (sum of rectus femoris, vastus lateralis and vastus Intermedius)	↔		
					Biopsy:			
					Vastus lateralis type I fibre muscle Thickness	↔		
					Vastus lateralis type II fibre muscle thickness	↔		
					**Strength:**			
					Combined force and load cell IMVC:			
					Leg press	↔		
Wikstrom-Frisen et al., 2017 [25]	**Group 1:** OCP-users: 11 Naturally menstruating: 8 **Group 2:** OCP-users: 10 Naturally menstruating: 9 **Group 3:** OCP-users: 11 Naturally menstruating: 10	Trained (*Mean resistance exercise training experience of 3.5 years*)	16	Yes	**Hypertrophy:**		High-frequency periodised leg resistance training during the first 2 weeks of the menstrual/OCP cycle was more beneficial in terms of power gain, strength gain and increased leg lean mass compared with high-frequency resistance training during the last 2 weeks	5
					DXA:			
					Leg lean mass	↔		
					Whole body lean mass	↔		
					**Power:**			
					Counter-movement jump	↔		
					Squat jump	↔		
					**Strength:**			
					Isokinetic dynamometer peak torque:			
					Single-leg knee extension (right and left)	↔		
					Single-leg knee flexion (right and left)	↔		

*CSA* cross-sectional area, *DXA* dual-energy X-ray absorptiometry, *IMVC* isometric maximal voluntary contraction, *MRI* magnetic resonance imaging, *NCAA* National Collegiate Athletic Association, *OCP* oral contraceptive pill, *RM* repetition maximum

↑ = significant difference favouring OCP group; ↔  = No significant difference between groups; ↓ = significant difference favouring OCP non-users group

*Shared participants

**Table 2:** Oral contraceptive pill (OCP) types and menstrual status descriptions from included studies comparing OCP users and non-users following matched resistance exercise training interventions

Study	Duration of OCP use mean ± SD	OCP type	OCP brand name	OCP dosage	Non-user criteria
Dalgaard et al., 2019	6.1 ± 5.0 years	Third-generation combined monophasic (*n* = 7)	Lindynette	75 μg gestodene and 30 μg ethinyl oestradiol	*n* = 14; had regular menstrual cycles (self-report) for at least 1 year (within the range of 24–35 days)
		Third-generation combined monophasic (*n* = 5)	Gestonette	75 μg gestodene and 20 μg ethinyl oestradiol	
		Third-generation combined monophasic (*n* = 2)	Novynette	150 μg desogestrel and 20 μg ethinyl oestradiol	
Dalgaard et al., 2022*	6.5 ± 2.5 years	Second-generation combined monophasic (*n* = 17)	Femicept	150 μg levonorgestrel and 30 μg ethinyl oestradiol	*n* = 18; had not been using any form of hormonal contraceptive for the past 3 months and reported that they had experienced a regular menstrual cycle (menses every 24–35 days) at last 3 months or more before the intervention
		Second-generation combined monophasic (*n* = 3)	Cilest	250 μg norgestimate and 35 μg ethinyl oestradiol	
Nichols et al., 2008	NR	Second-generation combined (*n* = 13)	NR	NR	*n* = 18; had regular menstrual cycles (menses every 25–35 days)
Oxfeldt et al., 2020*	6.5 ± 2.5 years	Second-generation combined monophasic (*n* = 17)	Femicept	150 μg levonorgestrel and 30 μg ethinyl oestradiol	*n* = 18; had not been using any form of hormonal contraceptive for the past 3 months and reported that they had experienced a regular menstrual cycle (menses every 24–35 days) at last 3 months or more before the intervention
		Second-generation combined monophasic (*n* = 3)	Cilest	250 μg norgestimate and 35 μg ethinyl oestradiol	
Reichman and Lee, 2021	NR	Oral contraceptives (*n* = 34)	Orthotricycle, Orthonovum, Orthocept, Desogen, Nordette, Alesse, Tivora, Estrostep, Loestrin	NR	*n* = 38; no information was available on the menstrual status of OCP non-users
Romance et al., 2019	NR	Second-generation (*n* = 4)	NR	150 μg levonorgestrel and 30 μg ethinyl oestradiol	*n* = 11; had reported to have regular menstrual cycles (i.e. occurring on a 28- to 30-day cycle) and had not taken any form of synthetic oestrogen or progesterone for at least 6 months prior to the study
		Third-generation (*n* = 8)	NR	50 μg gestodene and 30 μg ethinyl oestradiol	
Sung et al., 2022	≥ 1 year prior to study	Second-generation combined monophasic (*n* = 3)	Anastrella 28	150 μg levonorgestrel and 30 μg ethinyl oestradiol	*n* = 40; had no OCP use or hormonal treatment in the past year; 2 months of monitoring daily body temperature were performed to establish regularity of menstrual cycle prior to beginning intervention. Average menstrual cycle duration throughout intervention was 28.2 ± 1.2 days
		Second-generation combined monophasic (*n* = 10)	Cilest	250 μg norgestimate and 35 μg ethinyl oestradiol	
		Second-generation combined monophasic (*n* = 8)	Femicept	150 μg levonorgestrel and 30 μg ethinyl oestradiol	
		Second-generation combined monophasic (*n* = 9)	Loette	100 μg levonorgestrel and 20 μg ethinyl oestradiol	
		Second-generation combined monophasic (*n* = 4)	Microstad	150 μg levonorgestrel and 30 μg ethinyl oestradiol	
Wikstrom-Frisen et al., 2017	NR	Combined monophasic (*n* = 11)	NR	NR	*n* = 44; had a regular menstrual cycle of 28 days (acceptable 21–35 days)
		Combined triphasic (*n* = 20)	NR	NR	

*NR* not reported

*Shared participants

The inter-rater agreement statistics support strong agreement between authors. Initially the absolute agreement between the two first authors for all extracted continuous data using the two-way mixed effect model and “single rater” unit for ICC was 0.99 [0.99–0.99], *p* < 0.001. The initial inter-rater reliability for moderator coding was in perfect agreement (unweighted Cohen’s kappa [2, 330] = 1.00, *z* = 0, *p* < 0.001, 95% CI = [1.00, 1.00]; percent agreement = 100%).

### Hypertrophy Outcomes

For hypertrophy outcomes, 65% of the outcome estimates were positive (favouring the OCP condition), ranging from − 0.39 to 1.25. The multivariate model indicated that the standardised mean change difference between the conditions was 0.01 (95% CI [− 0.11, 0.13], *t* = 0.14,* p* = 0.90). The standardised mean change difference did not differ significantly from zero and showed no evidence of between-study heterogeneity (*χ*^2^(1) = 0.00, *p* = 1.00, *τ*^2^_between-studies_ = 0.00, *τ*^2^_within-studies_ = 0.09, *I*^2^_between-studies_ = 0%, *I*^2^_within-studies_ = 52.3%).

**Fig. 2:**
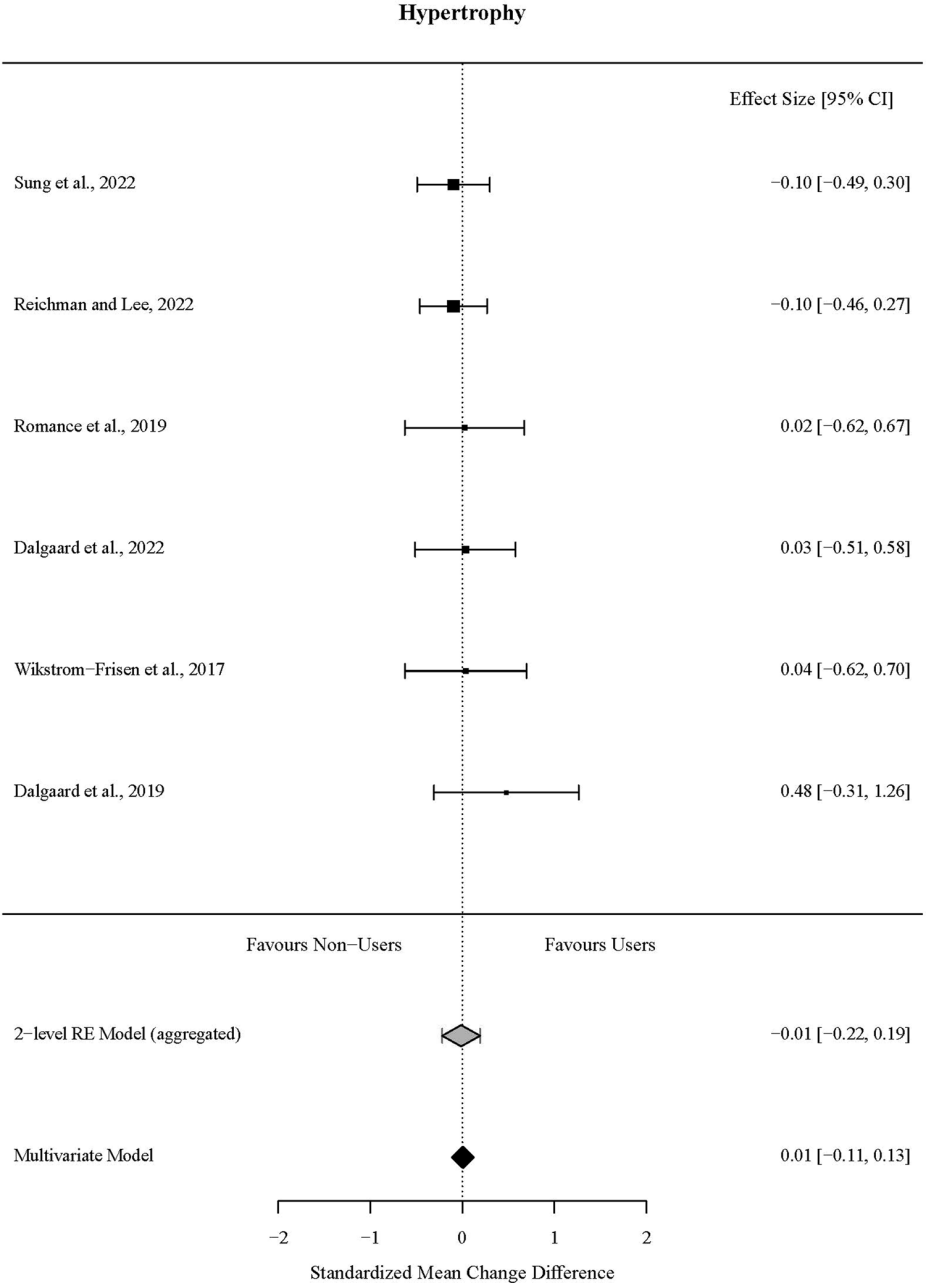
Forest plot of skeletal muscle hypertrophy outcomes from included studies comparing oral contraceptive pill (OCP) users and OCP non-users following matched resistance exercise training interventions

The effect sizes aggregated at the study level (one effect per study displayed per outcome) and their CIs, as well as the standardised mean change difference according to a meta-analytic multivariate model and two-level random effects model, are displayed in Fig. 2. Individual effects sizes and their CIs are shown in Electronic Supplementary File Fig. S4. Influential studies and outlier analyses are shown in Supplementary Figs. S2 and S3, respectively.

### Power Outcomes

**Fig. 3:**
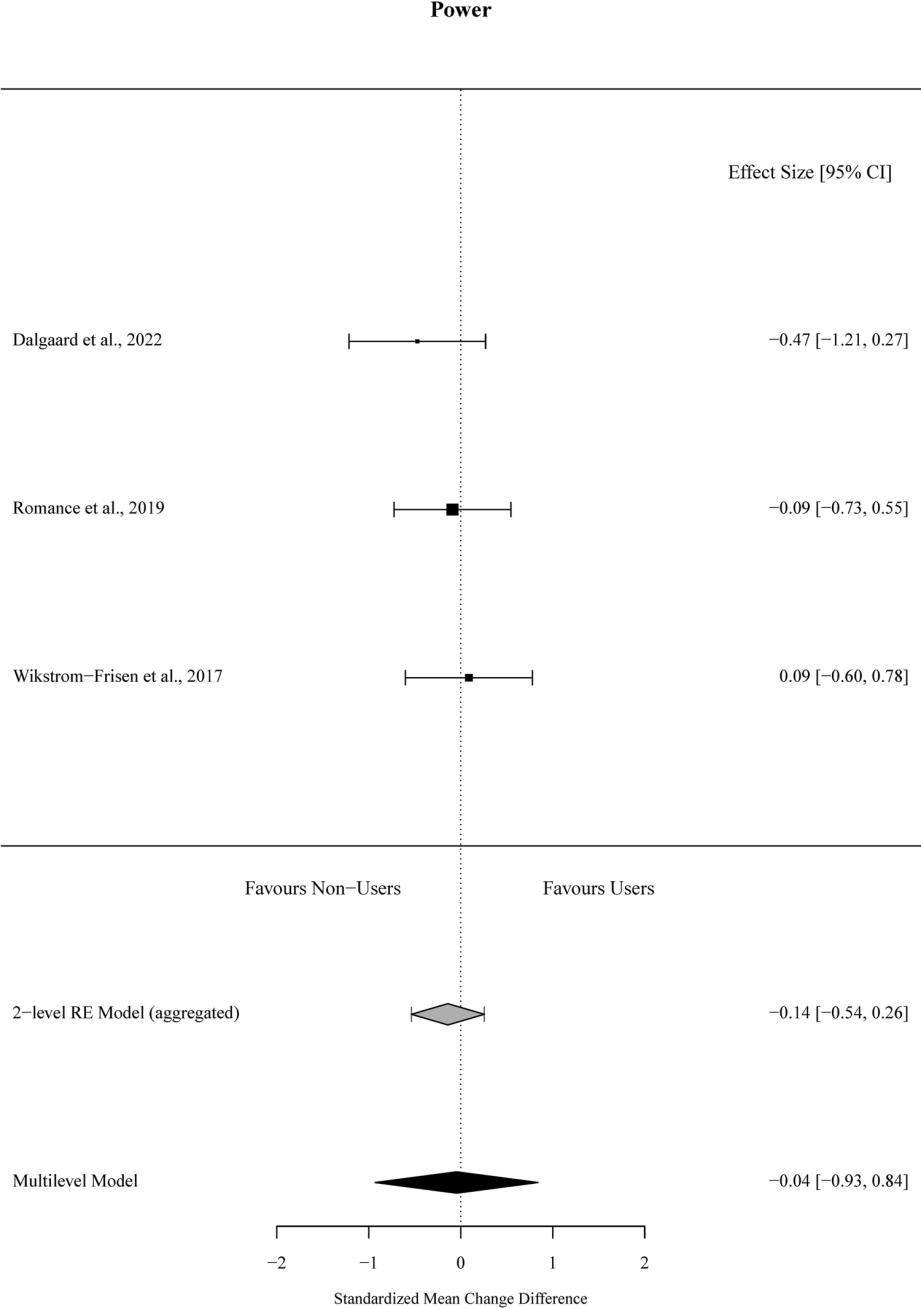
Forest plot of skeletal muscle power outcomes from included studies comparing oral contraceptive pill (OCP) users and OCP non-users following matched resistance exercise training interventions

For power outcomes, 37.5% of the outcome estimates were positive (favouring the OCP condition), ranging from − 0.47 to 0.38. The multivariate model indicated that the standardised mean change difference between the conditions was − 0.04 (95% CI [− 0.93, 0.84], *t* =  − 0.29,* p* = 0.80). The standardised mean change difference did not differ significantly from zero, and there was no between-study heterogeneity (*χ*^2^(1) = 0.00, *p* = 1.00, *τ*^2^_between-studies_ = 0.00, *τ*^2^_within-studies_ = 0.04, *I*^2^_between-studies_ = 0%, *I*^2^_within-studies_ = 21.3%). The effect sizes aggregated at the study level (one effect per study displayed per outcome) and their CIs, as well as the standardised mean change difference according to a meta-analytic multivariate model and two-level random effects model, are displayed in Fig. 3. Individual effects sizes and their CIs are shown in Electronic Supplementary File Fig. S4. Influential studies and outlier analyses are shown in Supplementary Figs. S2 and S3, respectively.

### Strength Outcomes

**Fig. 4:**
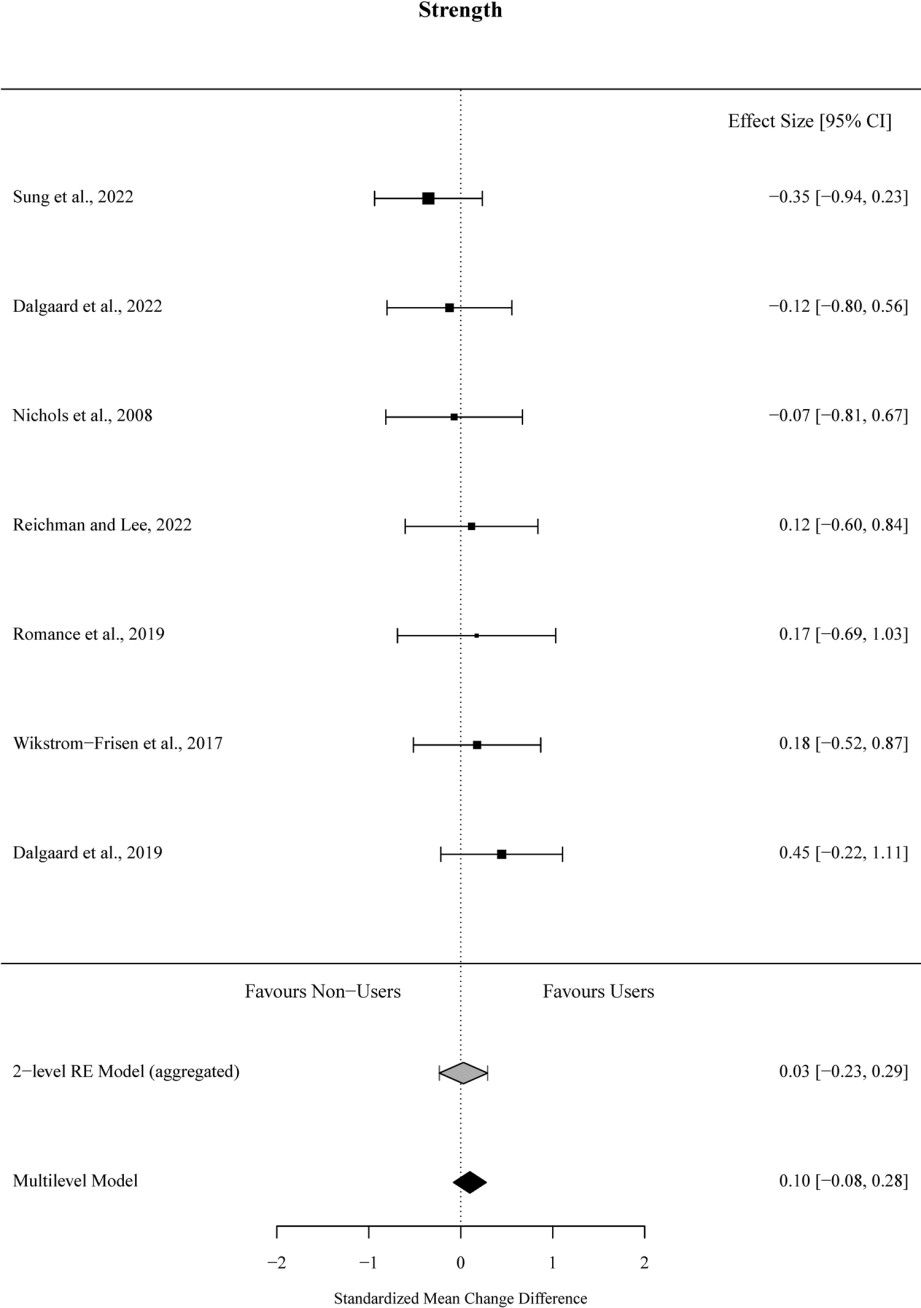
Forest plot of skeletal muscle strength outcomes from included studies comparing oral contraceptive pill (OCP) users and OCP non-users following matched resistance exercise training interventions

For strength outcomes, 62% of the outcome estimates were positive (favouring the OCP condition), ranging from − 0.35 to 0.62. The multivariate model indicated that the standardised mean change difference between the conditions was 0.10 (95% CI [− 0.08, 0.28], *t* = 1.48,* p* = 0.20). The standardised mean change difference did not differ significantly from zero and showed no between-study heterogeneity (*χ*^2^(1) = 0.00, *p* = 1.00, *τ*^2^_between-studies_ = 0.00*, τ*^2^_within-studies_ = 0.03, *I*^2^_between-studies_ = 0%, *I*^2^_within-studies_ = 18.8%). The effect sizes aggregated at the study level (one effect per study displayed per outcome) and their CIs, as well as the standardised mean change difference according to a meta-analytic multivariate model and two-level random effects model, are displayed in Fig. 4. Individual effects sizes and their CIs are shown in Electronic Supplementary File Fig. S4. Influential studies and outlier analyses are shown in Supplementary Figs. S2 and S3, respectively.

### Sensitivity Analyses

The sensitivity analyses were computed using alternative pre–post correlations in computing the effect sizes as well as examining different autocorrelations in computing the variance–covariance matrix. The results of the sensitivity analyses are listed in Table 3.

**Table 3:** Sensitivity analysis of multilevel meta-analyses comparing oral contraceptive pill (OCP) users and OCP non-users following matched resistance exercise training interventions using alternative values for pre-test–post-test correlations and correlation matrices

Sensitivity analysis procedure	Hypertrophy outcomes						Power outcomes						Strength outcomes					
	ES	95% CI	*τ*^2^_within-studies_	*τ*^2^_between-studies_	*I*^2^_within-studies_	*I*^2^_between-studies_	ES	95% CI	*τ*^2^_within-studies_	*τ*^2^_between-studies_	*I*^2^_within-studies_	*I*^2^_between-studies_	ES	95% CI	*τ*^2^_within-studies_	*τ*^2^_between-studies_	*I*^2^_within-studies_	*I*^2^_between-studies_
0.7 pre–post correlation and 0.9 correlation matrix	0.01	[− 0.11, 0.13]	0.09	0.00	52.28	0.00	− 0.04	[− 0.93, 0.84]	0.04	0.00	21.30	0.00	0.10	[− 0.08, 0.28]	0.03	0.00	18.77	0.00
0.7 pre–post correlation and 0.7 correlation matrix	0.01	[− 0.12, 0.13]	0.07	0.00	44.15	0.00	− 0.04	[− 0.91, 0.83]	0.01	0.00	4.51	0.00	0.09	[− 0.10, 0.28]	0.01	0.00	5.49	0.00
0.7 pre–post correlation and 0.5 correlation matrix	0.01	[− 0.13, 0.14]	0.05	0.00	36.40	0.00	− 0.04	[− 0.92, 0.83]	0.01	0.00	4.51	0.00	0.09	[− 0.10, 0.28]	0.01	0.00	5.49	0.00
0.5 pre–post correlation and 0.9 correlation matrix	0.00	[− 0.12, 0.11]	0.09	0.00	40.66	0.00	− 0.06	[− 0.96 0.83]	0.03	0.00	12.18	0.00	0.09	[− 0.10, 0.29]	0.02	0.00	11.45	0.00
0.5 pre–post correlation and 0.7 correlation matrix	0.00	[− 0.13, 0.12]	0.06	0.00	30.75	0.00	− 0.06	[− 0.96, 0.84]	0.00	0.00	0.00	0.00	0.07	[− 0.14, 0.28]	0.00	0.00	0.00	0.00
0.5 pre–post correlation and 0.5 correlation matrix	0.00	[− 0.13, 0.13]	0.04	0.00	22.09	0.00	− 0.06	[− 0.96, 0.84]	0.00	0.00	6.46	3.24	0.07	[− 0.14, 0.28]	0.00	0.00	0.00	0.00
0.9 pre–post correlation and 0.9 correlation matrix	0.02	[− 0.11, 0.15]	0.09	0.00	72.20	0.00	− 0.02	[− 0.90, 0.87]	0.04	0.00	43.80	0.00	0.11	[− 0.06, 0.27]	0.04	0.00	37.56	0.00
0.9 pre–post correlation and 0.7 correlation matrix	0.02	[− 0.11, 0.15]	0.07	0.00	68.02	0.00	− 0.02	[− 0.88, 0.84]	0.03	0.00	33.66	2.91	0.10	[− 0.07, 0.28]	0.25	0.00	29.66	0.00
0.9 pre–post correlation and 0.5 correlation matrix	0.02	[− 0.11, 0.16]	0.06	0.00	63.45	0.00	− 0.03	[− 0.84, 0.79]	0.01	0.01	19.82	9.93	0.10	[− 0.08, 0.28]	0.02	0.00	21.11	0.00

## Discussion

This is the first meta-analysis to investigate the influence of HC use on skeletal muscle hypertrophy, power and strength adaptations in response to resistance exercise training, and found that OCP use had no statistically significant effect on any of these adaptations. Based on the present analysis, there is no evidence-based rationale to advocate for or against the use of OCPs in females partaking in resistance exercise training to increase hypertrophy, power and/or strength, nor is there evidence that HC use would attenuate these adaptations. Rather, an individualised approach, considering an individual’s response to OCPs, and their reason(s) for use may be more appropriate.

There are several suggested mechanisms by which sex hormones may influence adaptations to resistance exercise training. OCPs downregulate the endocrine production of the primary ovarian hormones, i.e., oestrogen and progesterone. Oestrogen may influence pathways and processes that influence muscular adaptations to resistance exercise training (i.e. protein turnover, myosin function and satellite cell activity), but its role in the regulation of muscle mass is unclear, and potential mechanisms mediated by progesterone are largely unknown [38]. Oestrogen likely plays a role in modulating protein synthesis/degradation pathways, with differing protein synthesis rates observed in post-menopausal females undergoing oestrogen replacement therapy, compared with those not undergoing hormone replacement therapy [39, 40]. Oestrogen may influence muscle strength, via its influence on myosin proteins, as demonstrated by oestrogen deficiency (observed in rodent models and during menopause), negatively impacting the structure–function relation of myosin and actin during activity, reducing force-generating capacity and increasing fatiguability [41]. Oestrogen may also influence satellite cell activity and function by modulating paired box homeotic gene 7 (a marker of satellite cell number), myogenic differentiation factor D-positive fibres (a transcription factor involved in the activation of muscle-specific genes, leading to the differentiation of myoblasts into mature muscle fibres) and DNA uptake of bromo-deoxyuridine (an indicator of muscle cell proliferation). Yet these effects are predominantly shown in ovariectomised rodent models receiving oestrogen replacement [42], with very few studies in humans [43], and further investigation is warranted. Of note is that exogenously administered synthetic sex hormones may not be bioidentical to endogenous sex hormones, and therefore may not exert the same effect as endogenous oestrogen and progesterone [44, 45]. Given the potentially different hormonal profile experienced by OCP users, i.e., downregulated endogenous levels of oestrogen and progesterone, it could be argued that OCP users may not benefit from theoretical positive benefits of endogenous oestrogen for skeletal muscle adaptations to resistance exercise training. However, the present analysis does not support this hypothesis.

Some methodological considerations that are important to contextualise the findings of this study warrant further discussion. The average study duration was 11.6 weeks, yet increasing lean body mass through targeted interventions is a relatively slower process compared with muscular strength. On average, muscular strength increases by 25% in females following 15-weeks of resistance exercise training, while lean body mass increases by 3.3% (1.4 kg) in the same period [46]. Differences in skeletal muscle hypertrophy, if any, between OCP users and non-users may require a longer time course to manifest than has been studied to date. Of the included studies, approximately half included untrained participants, which may influence the magnitude of response observed in the individual outcomes measures in a given time frame, and may also limit extrapolations to trained or athletic populations. Indeed future studies may wish to investigate whether training status would be a moderator of the influence, if any, of HC use on exercise-training-induced adaptations. Studies had relatively small sample sizes (mean *n* = 45; range 28–74), with mean group sizes of 17 across all measures. Larger sample sizes may be warranted in future studies, as the magnitude of response to resistance exercise training varies extensively between individuals for both hypertrophy (− 11–30%) and strength (− 8–60%) outcomes [47]. Only three of the studies [19, 23, 25] that met the inclusion criteria reported power outcomes (all lower body), providing only eight effects, resulting in the meta-analysis model reported likely being underpowered for this outcome measure, evident by the wide confidence interval reported. Therefore, the results of the meta-analysis of the power outcomes should be interpreted with caution. Several studies grouped participants using various brands (differing formulations and dosages), and in some cases, differing generations (differing progestin components). Grouping participants using different types of OCP results in various concentrations of endogenous sex hormones and could result in non-homogenous participant groups [48]. As the potential impact, if any, of HCs on adaptation are likely mediated predominantly by the oestrogenic component, grouping participants using OCPs of differing dosages, androgenicity or using progestin-only pills is problematic. Genetic variations in tissue-specific oestrogen sensitivity also exist which may confound any potential influence of different contraceptive types [49]. Menstrual cycle status was predominantly confirmed through self-report measures, which is notable because anovulatory cycles and oestrogen deficiency can occur despite regular menstruation [50], and the effect of this, if any, on exercise-training-induced adaptations remains to be fully determined.

OCPs are used not only to prevent pregnancy, but for multiple reasons, such as in athletes for the alleviation of menstrual-related symptomatology and manipulation of the bleeding phase [51, 52]. Negative menstrual cycle symptomology is often reported as a barrier to engaging in exercise training, resulting in reduced training frequency, intensity and volume [53]. In theory, if OCP use reduced the negative aspects of menstrual cycle-related symptomatology on exercise performance, resistance exercise training adaptations may be enhanced in these individuals by facilitating the completion of higher frequencies, intensities and volume of training. Based on the present analysis, it must be stressed that at present there is no evidence-based rationale to advocate for or oppose the use of OCPs in females participating in resistance exercise training when aiming to increase hypertrophy, power and/or strength.

Future research should consider longitudinal analysis using sufficient sample sizes to account for large variability in exercise response [54], as differences, if any, between HC users and non-users may take considerable time to manifest, particularly for hypertrophy. The studies published to date investigating the influence of HCs on adaptations to resistance exercise training have exclusively investigated OCPs. Research should also examine the potential influence of different HC types (injection, intrauterine devices, implants, etc.), which result in differing hormonal profiles, on adaptations to resistance exercise training. In future investigations examining the influence of OCPs specifically, appropriate levels of detail describing the type of OCP and providing appropriate biochemical outcomes, such as blood samples, are needed to confirm the hormonal profiles of OCP users and non-users, as previously advocated [12, 55]. However, currently available blood analysis techniques only measure endogenous oestradiol levels, and do not allow for appropriate measurement of exogenous synthetic ethinyl oestradiol. Future research should also ensure intervention groups are appropriately designed to minimise the grouping of OCP users who use different OCP types and dosages, or adequately account for these subgroup differences in their statistical analysis. The impact, if any, of HC and OCPs on power-related adaptations are understudied and warrant further investigation.

## Conclusion

This systematic review is the first to employ meta-analysis to conduct a between-group comparison of skeletal muscle hypertrophy, power and strength adaptations to resistance exercise training in HC users and non-users. The main findings were that OCPs were the only HC studied to date, and OCP use had no statistically significant effect on these adaptations in response to resistance exercise training interventions of ~ 12 weeks in duration. As such, these data to date suggest that OCP use does not positively or negatively influence hypertrophy, power or strength adaptations in females partaking in resistance exercise training.

### Supplementary Information

Below is the link to the electronic supplementary material.

Supplementary file1 (DOCX 545 KB)

## Declarations

### Availability of Data and Material

The aggregated dataset used for the analysis can be found on the OSF website (https://osf.io/wumav/?view_only=8ed6db48dcad465ba36fcc95fb6d3ee7). Additional information can be shared on request.

### Coder Availability

The R-code used for the analysis can be found on the OSF website (https://osf.io/wumav/?view_only=8ed6db48dcad465ba36fcc95fb6d3ee7). Additional information can be shared on request.
