# Supplementary material for: The Effect of Hormonal Contraceptive Use on Skeletal Muscle Hypertrophy, Power and Strength Adaptations to Resistance Exercise Training: A Systematic Review and Multilevel Meta-analysis
Source: Sports Med. 2023 Sep 27;54(1):105–25. doi: 10.1007/s40279-023-01911-3 (PMC10798924; doi:10.1007/s40279-023-01911-3)
Supplement: Supplementary file 1 — Supplementary file1 (DOCX 545 KB) [file 40279_2023_1911_MOESM1_ESM.docx]

**Electronic Supplementary Material**

**Nolan *et al*. The effect of oral contraceptive use on hypertrophy, power, and strength adaptations to resistance exercise training: a systematic review and multilevel meta-analysis.**

*Sports Medicine*

DOI : 10.1007/s40279-023-01911-3.

Corresponding author: Dr. Brendan Egan, School of Health and Human Performance, Dublin City University, Dublin, Ireland. Email: [brendan.egan@dcu.ie](mailto:brendan.egan@dcu.ie)

**Electronic Supplementary Material Table S1. PRISMA Checklist for the study titled “The effect of oral contraceptive use on hypertrophy, power, and strength adaptations to resistance exercise training: a systematic review and multilevel meta-analysis”**

| **Section and Topic** | **Item #** | **Checklist item** | **Location where item is reported** |
| --- | --- | --- | --- |
| **TITLE** | | |  |
| Title | 1 | Identify the report as a systematic review. | 1 |
| **ABSTRACT** | | |  |
| Abstract | 2 | See the PRISMA 2020 for Abstracts checklist. | 1 |
| **INTRODUCTION** | | |  |
| Rationale | 3 | Describe the rationale for the review in the context of existing knowledge. | 2 |
| Objectives | 4 | Provide an explicit statement of the objective(s) or question(s) the review addresses. | 2 |
| **METHODS** | | |  |
| Eligibility criteria | 5 | Specify the inclusion and exclusion criteria for the review and how studies were grouped for the syntheses. | 3-4 |
| Information sources | 6 | Specify all databases, registers, websites, organisations, reference lists and other sources searched or consulted to identify studies. Specify the date when each source was last searched or consulted. | 2 |
| Search strategy | 7 | Present the full search strategies for all databases, registers and websites, including any filters and limits used. | 2 |
| Selection process | 8 | Specify the methods used to decide whether a study met the inclusion criteria of the review, including how many reviewers screened each record and each report retrieved, whether they worked independently, and if applicable, details of automation tools used in the process. | 5 |
| Data collection process | 9 | Specify the methods used to collect data from reports, including how many reviewers collected data from each report, whether they worked independently, any processes for obtaining or confirming data from study investigators, and if applicable, details of automation tools used in the process. | 4-5 |
| Data items | 10a | List and define all outcomes for which data were sought. Specify whether all results that were compatible with each outcome domain in each study were sought (e.g. for all measures, time points, analyses), and if not, the methods used to decide which results to collect. | 4 |
|  | 10b | List and define all other variables for which data were sought (e.g. participant and intervention characteristics, funding sources). Describe any assumptions made about any missing or unclear information. | 4 |
| Study risk of bias assessment | 11 | Specify the methods used to assess risk of bias in the included studies, including details of the tool(s) used, how many reviewers assessed each study and whether they worked independently, and if applicable, details of automation tools used in the process. | 4-5 |
| Effect measures | 12 | Specify for each outcome the effect measure(s) (e.g. risk ratio, mean difference) used in the synthesis or presentation of results. | 4 |
| Synthesis methods | 13a | Describe the processes used to decide which studies were eligible for each synthesis (e.g. tabulating the study intervention characteristics and comparing against the planned groups for each synthesis (item #5)). | 3-4 |
|  | 13b | Describe any methods required to prepare the data for presentation or synthesis, such as handling of missing summary statistics, or data conversions. | 3-4 |
|  | 13c | Describe any methods used to tabulate or visually display results of individual studies and syntheses. | 5 |
|  | 13d | Describe any methods used to synthesize results and provide a rationale for the choice(s). If meta-analysis was performed, describe the model(s), method(s) to identify the presence and extent of statistical heterogeneity, and software package(s) used. | 4-5 |
|  | 13e | Describe any methods used to explore possible causes of heterogeneity among study results (e.g. subgroup analysis, meta-regression). | 4-5 |
|  | 13f | Describe any sensitivity analyses conducted to assess robustness of the synthesized results. | 4-5 |
| Reporting bias assessment | 14 | Describe any methods used to assess risk of bias due to missing results in a synthesis (arising from reporting biases). | 5 |
| Certainty assessment | 15 | Describe any methods used to assess certainty (or confidence) in the body of evidence for an outcome. | 5 |
| **RESULTS** | | |  |
| Study selection | 16a | Describe the results of the search and selection process, from the number of records identified in the search to the number of studies included in the review, ideally using a flow diagram. | 3&5 |
|  | 16b | Cite studies that might appear to meet the inclusion criteria, but which were excluded, and explain why they were excluded. | N/A |
| Study characteristics | 17 | Cite each included study and present its characteristics. | 6-10 |
| Risk of bias in studies | 18 | Present assessments of risk of bias for each included study. | Electronic Supplementary Materials |
| Results of individual studies | 19 | For all outcomes, present, for each study: (a) summary statistics for each group (where appropriate) and (b) an effect estimate and its precision (e.g. confidence/credible interval), ideally using structured tables or plots. | 16-18 |
| Results of syntheses | 20a | For each synthesis, briefly summarise the characteristics and risk of bias among contributing studies. | 6&13 |
|  | 20b | Present results of all statistical syntheses conducted. If meta-analysis was done, present for each the summary estimate and its precision (e.g. confidence/credible interval) and measures of statistical heterogeneity. If comparing groups, describe the direction of the effect. | 6&13 |
|  | 20c | Present results of all investigations of possible causes of heterogeneity among study results. | 6&13 |
|  | 20d | Present results of all sensitivity analyses conducted to assess the robustness of the synthesized results. | 6&13 |
| Reporting biases | 21 | Present assessments of risk of bias due to missing results (arising from reporting biases) for each synthesis assessed. | Electronic Supplementary Materials |
| Certainty of evidence | 22 | Present assessments of certainty (or confidence) in the body of evidence for each outcome assessed. | 6&13 |
| **DISCUSSION** | | |  |
| Discussion | 23a | Provide a general interpretation of the results in the context of other evidence. | 13 |
|  | 23b | Discuss any limitations of the evidence included in the review. | 13 & 17 |
|  | 23c | Discuss any limitations of the review processes used. | 17 |
|  | 23d | Discuss implications of the results for practice, policy, and future research. | 18-19 |
| **OTHER INFORMATION** | | |  |
| Registration and protocol | 24a | Provide registration information for the review, including register name and registration number, or state that the review was not registered. | 1 |
|  | 24b | Indicate where the review protocol can be accessed, or state that a protocol was not prepared. | 1 |
|  | 24c | Describe and explain any amendments to information provided at registration or in the protocol. | 1 |
| Support | 25 | Describe sources of financial or non-financial support for the review, and the role of the funders or sponsors in the review. | 19 |
| Competing interests | 26 | Declare any competing interests of review authors. | 19 |
| Availability of data, code and other materials | 27 | Report which of the following are publicly available and where they can be found: template data collection forms; data extracted from included studies; data used for all analyses; analytic code; any other materials used in the review. | 19 |

*From:*  Page MJ, McKenzie JE, Bossuyt PM, Boutron I, Hoffmann TC, Mulrow CD, et al. The PRISMA 2020 statement: an updated guideline for reporting systematic reviews. BMJ 2021;372:n71. doi: 10.1136/bmj.n71

For more information, visit: <http://www.prisma-statement.org/>

**Electronic Supplementary Material Table S2. TESTEX quality appraisal of included studies comparing oral contraceptive pill (OCP) users and non-OCP users following matched resistance exercise training interventions.**

| Study | Item 1. | Item 2. | Item 3. | Item 4. | Item 5a. | Item 5b. | Item 5c. | Item 6. | Item 7a. | Item 7b. | Item 8. | Item 9. | Item 10. | Item 11. | Total Score |
| --- | --- | --- | --- | --- | --- | --- | --- | --- | --- | --- | --- | --- | --- | --- | --- |
| Dalgaard et al., 2019 | 1 | 1 | 1 | 1 | 1 | 0 | 1 | 0 | 1 | 1 | 1 | 1 | 1 | 1 | **12** |
| Dalgaard et al., 2022 | 1 | 1 | 1 | 1 | 1 | 1 | 1 | 0 | 1 | 1 | 1 | 1 | 1 | 1 | **13** |
| Nichols et al., 2008 | 1 | 1 | 0 | 1 | 0 | 0 | 0 | 0 | 1 | 1 | 0 | 0 | 0 | 1 | **6** |
| Oxfeldt et al., 2020 | 1 | 1 | 1 | 1 | 1 | 1 | 1 | 0 | 1 | 1 | 1 | 1 | 1 | 1 | **13** |
| Reichmann and Lee, 2021 | 1 | 0 | 1 | 0 | 0 | 0 | 0 | 1 | 1 | 1 | 1 | 1 | 1 | 1 | **9** |
| Romance et al., 2019 | 1 | 1 | 1 | 0 | 0 | 0 | 0 | 0 | 1 | 1 | 1 | 1 | 1 | 1 | **9** |
| Sung et al., 2022 | 1 | 1 | 0 | 0 | 0 | 0 | 0 | 0 | 1 | 1 | 1 | 1 | 1 | 1 | **8** |
| Wikstrom-Frisen et al., 2017 | 1 | 0 | 1 | 0 | 0 | 0 | 0 | 0 | 1 | 1 | 1 | 0 | 0 | 0 | **5** |

| Item | Criteria |
| --- | --- |
| 1 | Eligibility criteria specified. |
| 2 | Groups defined and confirmed. |
| 3 | Groups similar at baseline. |
| 4 | Blinding of assessor (for at least one key outcome). |
| 5a | Study withdrawals reported. |
| 5b | Adverse events reported. |
| 5c | Session attendance report. |
| 6 | Intention-to-treat analysis. |
| 7a | Primary outcome reported. |
| 7b | Secondary outcome(s) reported. |
| 8 | Point measures and measures of variability for all reported outcome measures. |
| 9 | The type of hormonal contraceptive was described to the level of detail required for categorisation or replication. |
| 10 | Relative exercise intensity remained constant. |
| 11 | Exercise volume and energy expenditure. |

**Electronic Supplementary Material Table S3. Characteristics of the Resistance Exercise Training Interventions used in the studies analysed comparing oral contraceptive pill (OCP) users and non-OCP users following matched resistance exercise training interventions.**

| Study |  | Resistance Exercise Training Intervention |
| --- | --- | --- |
| Dalgaard et al., 2019 [20] |  | 10 weeks of progressive resistance exercise training performed 3 times per week supervised by physical therapists. Training intensities were estimated from the 1 RM test. The exercises consisted of seated knee extensions and inclined leg press performed in a progressive manner;  Week 1: 3x 12 repetitions of 15 RM  Week 2–3: 3x 12 repetitions of 12 RM  Week 4–5: 3x 10 repetitions of 10 RM  Weeks 6–10: 4x 10 repetitions of 10 RM. |
| Dalgaard et al., 2022* [19] |  | 10 weeks of supervised resistance exercise training with 3 sessions per week. Four different resistance-training exercises for the legs were performed: Leg press, leg curl, leg extension, and back extension. In addition, 2 upper body exercises were performed: Lat pull down and incline crunch. The length of one training session was 45 minutes. The exercises were performed in a progressive manner as 3–4 sets of 8–12 repetitions, corresponding to their 8–12 repetition maximum. Participants were encouraged to use maximal effort and train near momentary muscle failure.  All training sessions were supervised by an exercise therapist. The lifted loads were monitored using individual training logs to ensure subjects adjusted weights throughout the entire training period to maintain muscle loading as muscle strength increased. |
| Nichols et al., 2008 [22] |  | 12-week preseason strength development program. No attempt was made by the researchers to modify the athletes’ existing strength training program as designed by the university’s strength and conditioning coach. This program consisted of free weight and machine lifting exercises involving the major muscle groups, performed three times per week.  Resistance levels were based on percentages ranging from 50% to 80% of initial 1 repetition maximum tests. Individual training logs were maintained to monitor subjects’ numbers of sets, repetitions, resistance levels, and program compliance. |
| Oxfeldt et al., 2020* [15] |  | Same as Dalgaard et al., 2022 [19] above. |
| Reichmann and Lee, 2021 [16] |  | 10 weeks of resistance exercise training, performed 3 times a week under the supervision of exercise physiologists. The participants performed 3 sets of 6–10 repetitions for each of the following exercises with the resistance set at 75% of each individual’s predetermined maximum strength (chest press, incline press, lat pull-down, seated row, shoulder press, leg extension, hamstring curl, triceps extension, arm curl, back extension, and abdominal crunch. weight machines; leg press and calf raises were performed on a Universal leg press machine.  The subjects were instructed to perform as many repetitions as possible on every set until they reached muscle failure or achieved 10 repetitions with correct forms and full range of motion. For every participant, resistance settings were increased when a subject was able to complete 10 repetitions on a particular set. Resistance was adjusted as needed throughout the study according to the results of biweekly reviews of each subject’s progression. The resting time was 30 seconds between sets and one minute between exercises. |
| Romance et al., 2019 [23] |  | 3-week familiarization period to establish training loads for each exercise, followed by an 8-week intervention period.  All subjects performed the same exercises; bench press, barbell row, military press, lat pulldown, incline chest press, biceps curl and triceps pushdown, squat, lunge, leg press, hip thrust, leg extension, lying leg curl and standing calf raise. Subjects completed four training sessions per week. Training sessions were monitored by specialists, adjusting the loads whenever necessary. The lifted loads and perceived exertion in each exercise were monitored by the physical conditioning and strength specialist using a paper tracking form throughout the experiment. |
| Sung et al., 2022 [24] |  | 12 weeks of submaximal strength training. The initial training value was set to 85% of the maximal isometric strength test, and participants performed 3 sets of 8–12 repetitions on a leg-press machine with 2 min of resting intervals between sets until exhaustion. If any participant was able to perform more than 12 repetitions during a set, they increased the resistance by 10 kg (i.e., 85–95% of their maximal strength) to reduce the number of repetitions to 12 or less. Supervised training was performed 3 times a week using the leg-press machine and once a week at home using the participant’s own body weight (squats). For the latter exercise, participants performed 3 sets of 15–20 leg squats with 3–5 min of recovery between sets. |
| Wikstrom-Frisen et al., 2017 [25] |  | In this study of four menstrual/OCP cycles, periodised resistance training refers to high frequency leg training during two weeks of the menstrual cycle. The remaining two weeks of respective cycle the women performed the leg resistance training once a week as part of the training program.  In group 1 periodised high frequency training was performed (5 times per week) during the first two weeks of the menstrual cycle.  In group 2 periodised high frequency training was performed (5 times per week) during the last two weeks of the menstrual cycle.  The women in group 3 trained regularly (3 times per week) during the whole menstrual/OCP cycle.  The resistance training in the present study consisted of two exercises, leg press and leg curls, and were started at an individual load. The participants performed double leg press and leg curls three sets of 8-12 repetition maximum (RM) with 1-2 minutes of rest between sets and with 2-10 % increase in load applied when the individual performed the current workload for one or two repetitions over the desired number, according to recommendations to achieve strength gains.6, 7 The women also continued their ordinary training with the exception of leg exercises, which were exchanged to the exercise provided by the researchers. Throughout the study a gym instructor was available to guide the training. |

**
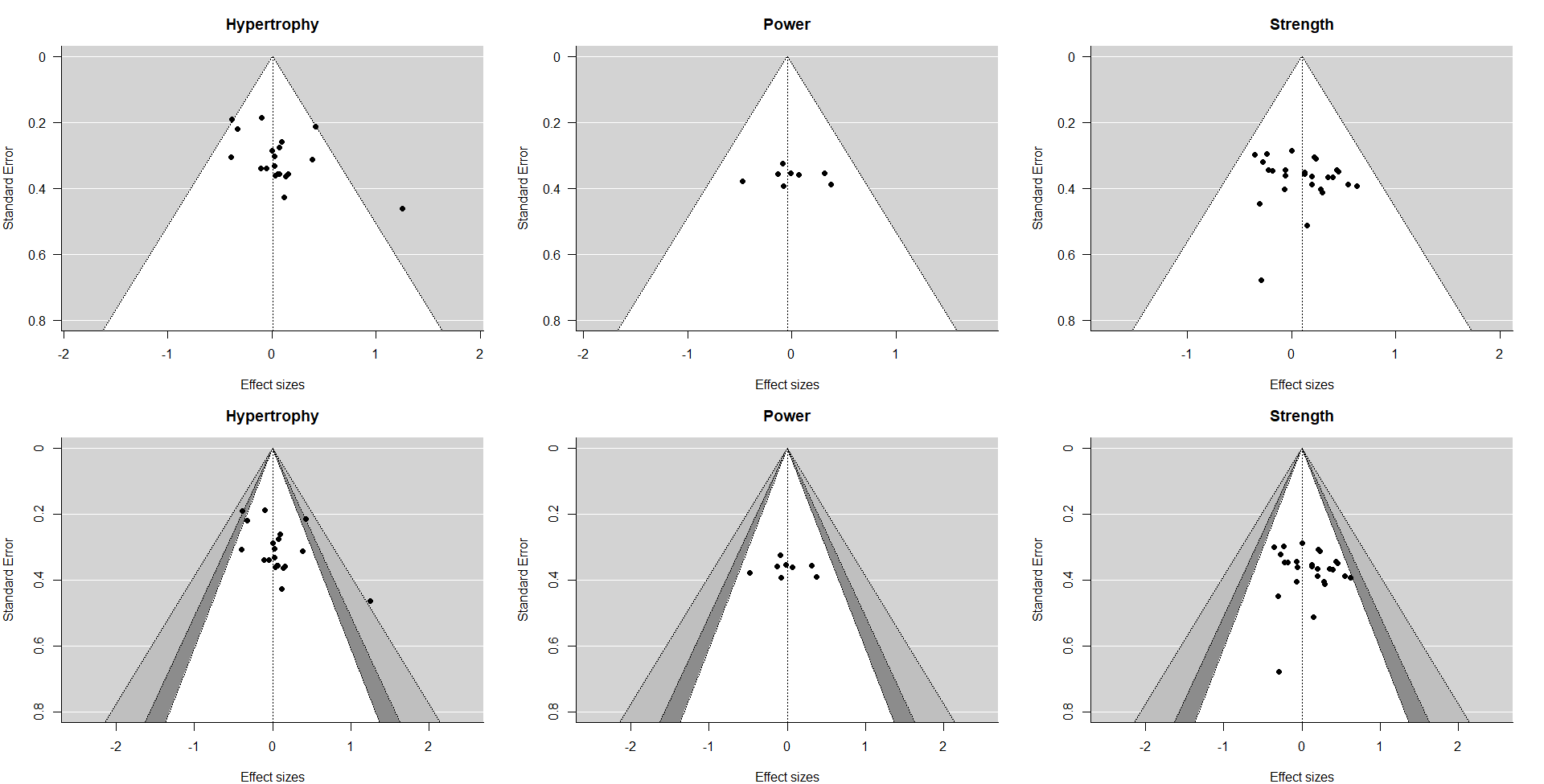
**

**Electronic Supplementary Material Figure S1 - Funnel plot publication bias analysis of included comparing oral contraceptive pill (OCP) users and non-OCP users following matched resistance exercise training interventions.**

**
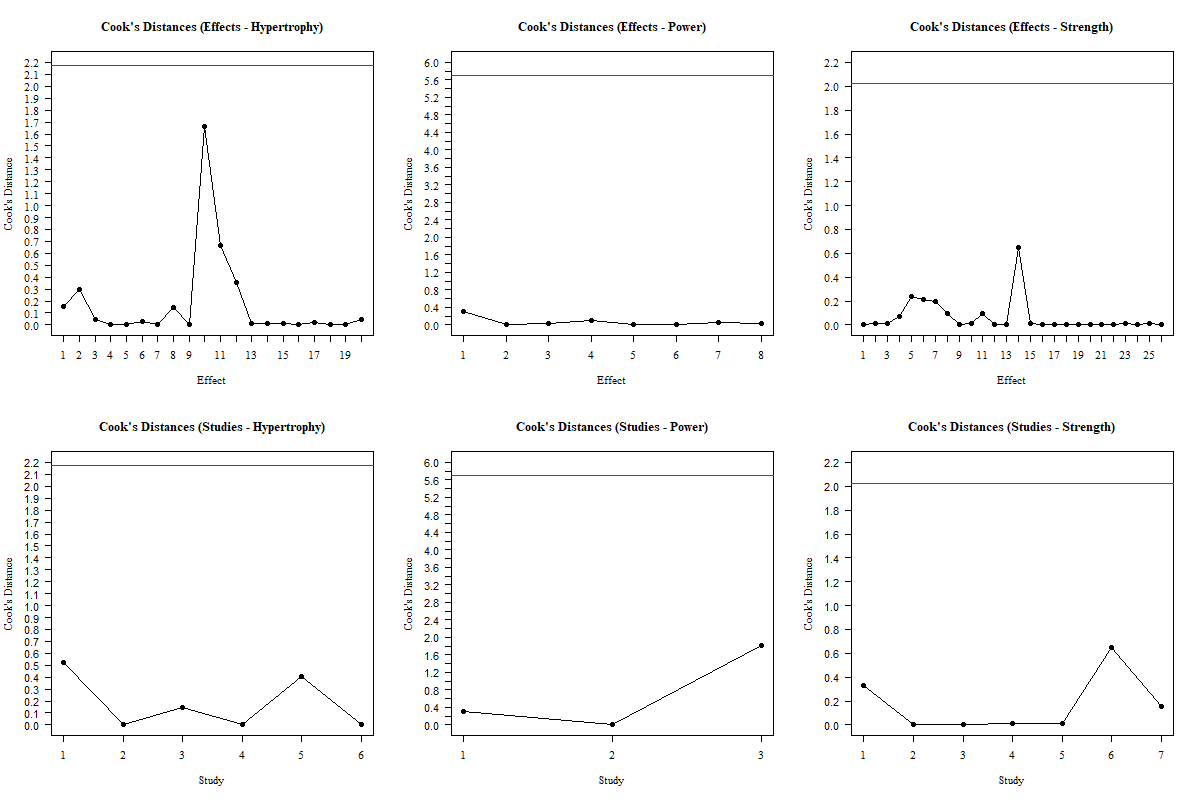
**

**Electronic Supplementary Material Figure S2. Influential effects and studies analysis from included studies comparing oral contraceptive pill (OCP) users and non-OCP users following matched resistance exercise training interventions.**

**
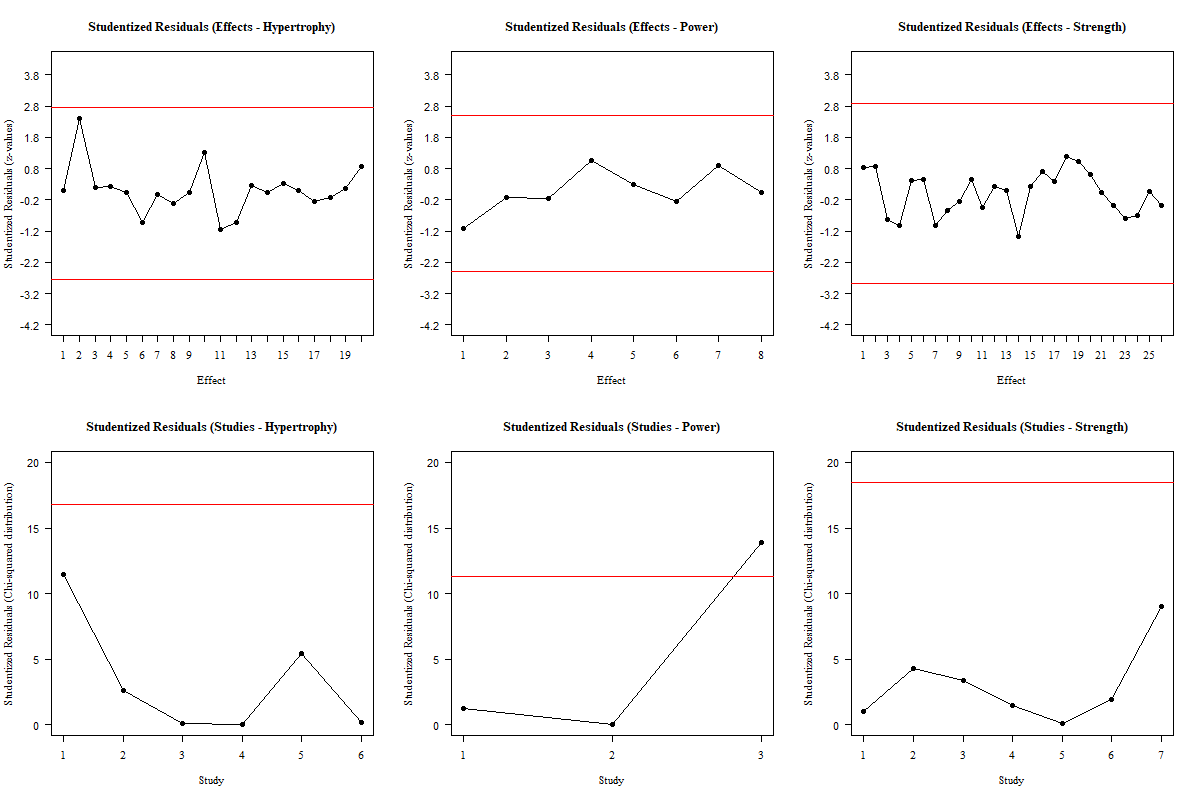
**

**Electronic Supplementary Material Figure S3. Outlier analysis of studies and individual effects from included studies comparing oral contraceptive pill (OCP) users and non-OCP users following matched resistance exercise training interventions.**


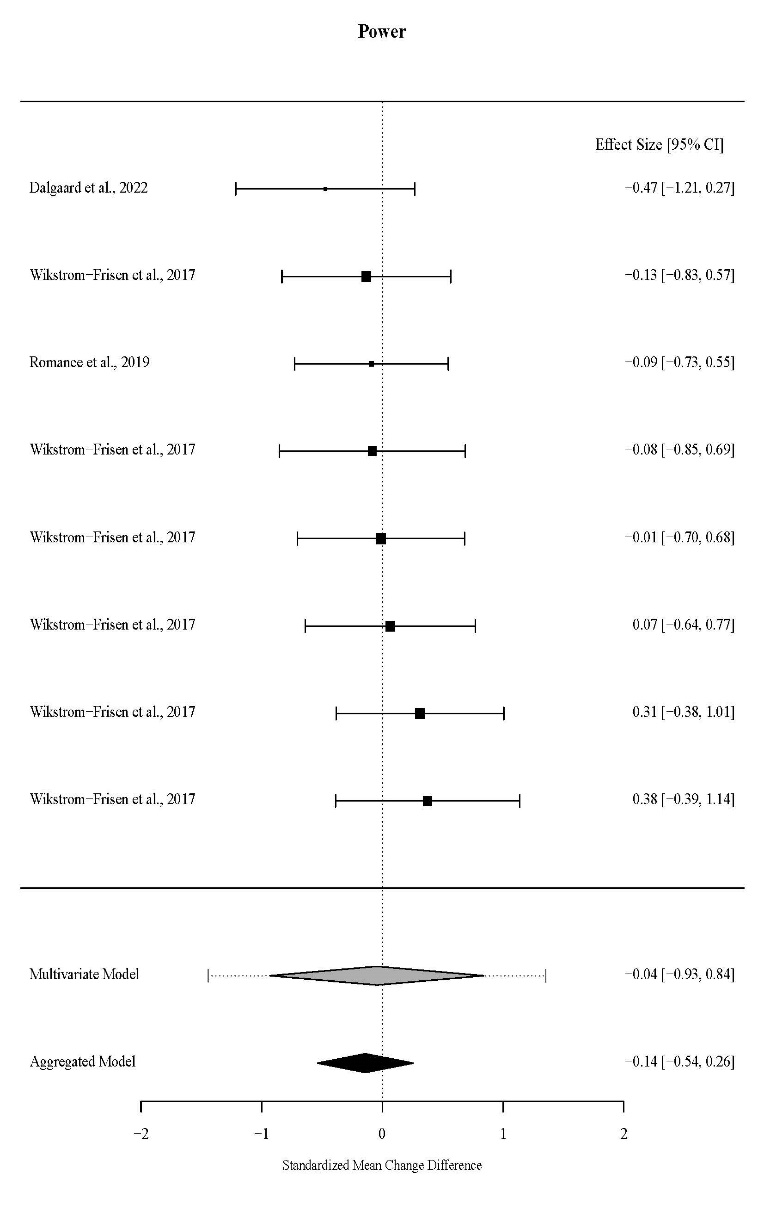

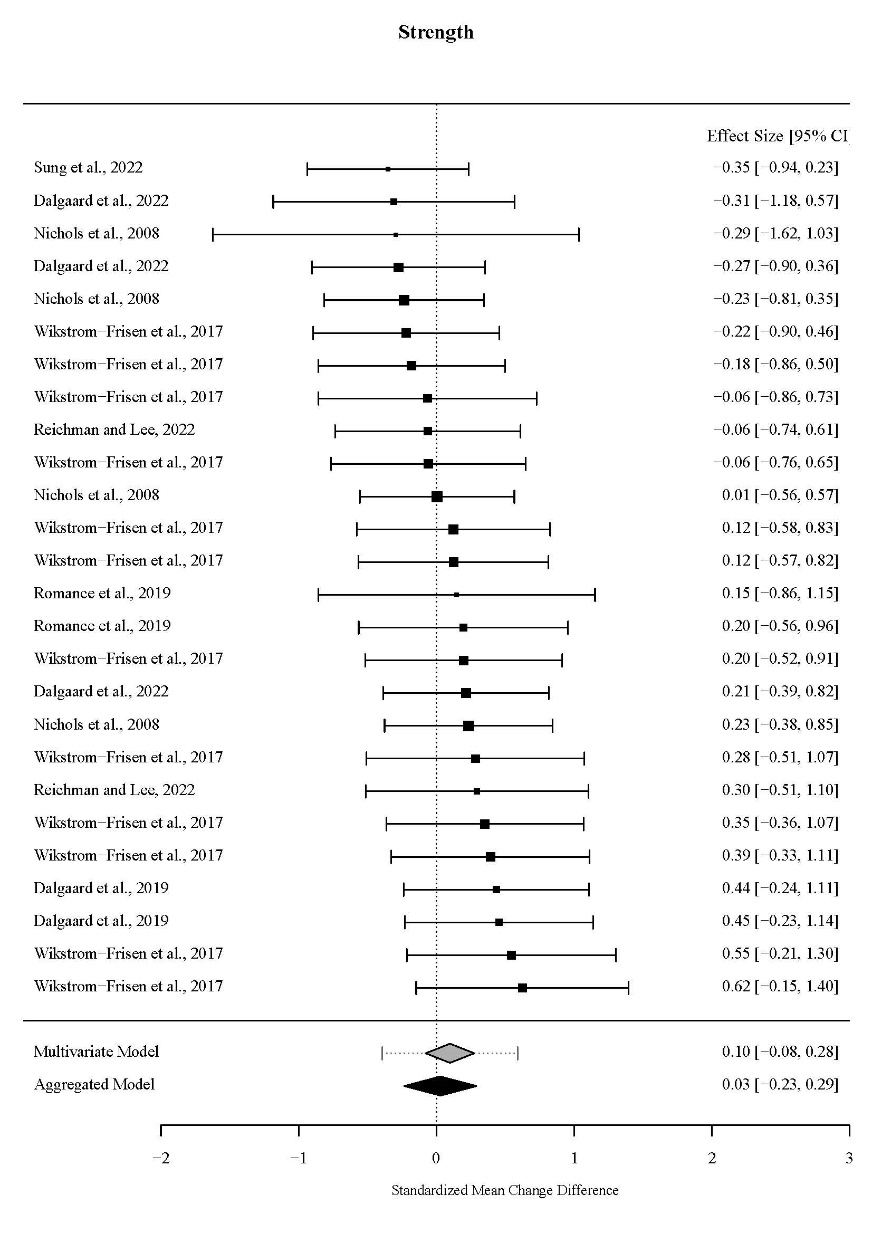

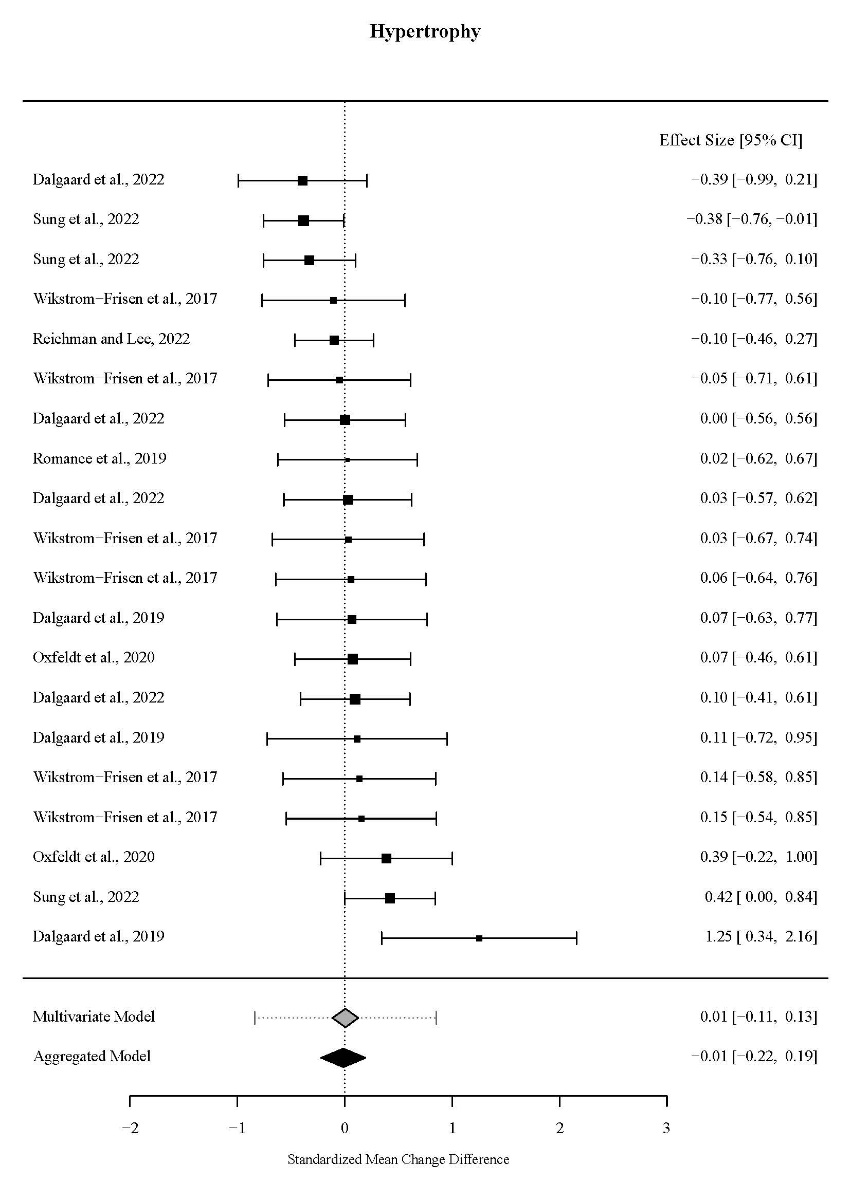


**Electronic Supplementary Material Figure S4. Forest Plots Displaying Individual Outcome Effects from included studies comparing oral contraceptive pill (OCP) users and non-OCP users following matched resistance exercise training interventions.**
